# Recommendations to overcome barriers to the use of artificial intelligence-driven evidence in health technology assessment

**DOI:** 10.3389/fpubh.2023.1088121

**Published:** 2023-04-26

**Authors:** Antal Zemplényi, Konstantin Tachkov, Laszlo Balkanyi, Bertalan Németh, Zsuzsanna Ida Petykó, Guenka Petrova, Marcin Czech, Dalia Dawoud, Wim Goettsch, Inaki Gutierrez Ibarluzea, Rok Hren, Saskia Knies, László Lorenzovici, Zorana Maravic, Oresta Piniazhko, Alexandra Savova, Manoela Manova, Tomas Tesar, Spela Zerovnik, Zoltán Kaló

**Affiliations:** ^1^Center for Health Technology Assessment and Pharmacoeconomics Research, Faculty of Pharmacy, University of Pécs, Pécs, Hungary; ^2^Syreon Research Institute, Budapest, Hungary; ^3^Department of Organization and Economics of Pharmacy, Faculty of Pharmacy, Medical University of Sofia, Sofia, Bulgaria; ^4^Medical Informatics R&D Center, Pannon University, Veszprém, Hungary; ^5^Department of Pharmacoeconomics, Institute of Mother and Child, Warsaw, Poland; ^6^Science Policy and Research Programme, Science Evidence and Analytics Directorate, National Institute for Health and Care Excellence (NICE), London, United Kingdom; ^7^Cairo University, Faculty of Pharmacy, Cairo, Egypt; ^8^Division of Pharmacoepidemiology and Clinical Pharmacology, Utrecht University, Utrecht, Netherlands; ^9^National Health Care Institute, Diemen, Netherlands; ^10^Basque Foundation for Health Innovation and Research, Barakaldo, Spain; ^11^Faculty of Mathematics and Physics, University of Ljubljana, Ljubljana, Slovenia; ^12^Syreon Research Romania, Tirgu Mures, Romania; ^13^G. E. Palade University of Medicine, Pharmacy, Science and Technology, Tirgu Mures, Romania; ^14^Digestive Cancers Europe, Brussels, Belgium; ^15^HTA Department of State Expert Centre of the Ministry of Health of Ukraine, Kyiv, Ukraine; ^16^National Council of Prices and Reimbursement of Medicinal Products, Sofia, Bulgaria; ^17^Department of Organisation and Management of Pharmacy, Faculty of Pharmacy, Comenius University in Bratislava, Bratislava, Slovakia; ^18^Ministry of Health, Ljubljana, Slovenia; ^19^Centre for Health Technology Assessment, Semmelweis University, Budapest, Hungary

**Keywords:** artificial intelligence—AI, machine learning, health technology assessment, evidence generation, Central and Eastern Europe

## Abstract

**Background:**

Artificial intelligence (AI) has attracted much attention because of its enormous potential in healthcare, but uptake has been slow. There are substantial barriers that challenge health technology assessment (HTA) professionals to use AI-generated evidence for decision-making from large real-world databases (e.g., based on claims data). As part of the European Commission-funded HTx H2020 (Next Generation Health Technology Assessment) project, we aimed to put forward recommendations to support healthcare decision-makers in integrating AI into the HTA processes. The barriers, addressed by the paper, are particularly focusing on Central and Eastern European (CEE) countries, where the implementation of HTA and access to health databases lag behind Western European countries.

**Methods:**

We constructed a survey to rank the barriers to using AI for HTA purposes, completed by respondents from CEE jurisdictions with expertise in HTA. Using the results, two members of the HTx consortium from CEE developed recommendations on the most critical barriers. Then these recommendations were discussed in a workshop by a wider group of experts, including HTA and reimbursement decision-makers from both CEE countries and Western European countries, and summarized in a consensus report.

**Results:**

Recommendations have been developed to address the top 15 barriers in areas of (1) human factor-related barriers, focusing on educating HTA doers and users, establishing collaborations and best practice sharing; (2) regulatory and policy-related barriers, proposing increasing awareness and political commitment and improving the management of sensitive information for AI use; (3) data-related barriers, suggesting enhancing standardization and collaboration with data networks, managing missing and unstructured data, using analytical and statistical approaches to address bias, using quality assessment tools and quality standards, improving reporting, and developing better conditions for the use of data; and (4) technological barriers, suggesting sustainable development of AI infrastructure.

**Conclusion:**

In the field of HTA, the great potential of AI to support evidence generation and evaluation has not yet been sufficiently explored and realized. Raising awareness of the intended and unintended consequences of AI-based methods and encouraging political commitment from policymakers is necessary to upgrade the regulatory and infrastructural environment and knowledge base required to integrate AI into HTA-based decision-making processes better.

## 1. Introduction

Sophisticated computational models and algorithms, combined with powerful computers and the availability of vast amounts of data, have recently accelerated the application of artificial intelligence (AI) in various fields. This is primarily driven by the development of machine learning (ML), which has contributed to advances in data science and statistical prediction (1).

Artificial intelligence has attracted much attention because of its potential in healthcare to improve access, quality and efficiency (2), but its adoption has been slow due to several factors that are specific to healthcare (e.g., legal and ethical restrictions to accessing patient level data, fragmented databases, interoperability issues with pooling data etc.) (2, 3), resulting in a lag of healthcare behind other industries (4). Although AI has been applied in several areas of healthcare, such as more accurate and faster detection, prediction, and diagnosis of diseases (5, 6), its use in supporting health policymaking has been limited so far (7–9). Nevertheless, some attempts have been made, especially during the COVID-19 pandemic crisis.^1^

Evidence-based decision-making and policymaking principles are widely accepted in developed health systems. Briefly, health technology assessment (HTA) is concerned with systematically evaluating the implications (direct and indirect) of adopting new health technologies and improving the evidence base for health policy decision-making. HTA is mainly used to support reimbursement decisions at the macro level to improve the efficient allocation of resources (10). Still, it can be used at the meso (hospital based HTA) or micro level (patient-professional decisions).

In recent years, HTA has become more reliant on healthcare systems’ data, such as claims data or electronic medical records, to generate real-world evidence that may inform provision or reimbursement decisions. To analyse and evaluate those data, different applications of AI have already been identified, including assessing the burden of illness, identifying drug utilization and patterns of use, generating patient-reported outcomes, evaluating the comparative effectiveness of interventions, and conducting economic evaluations (11). Specifically, ML methods could help enhance HTA by applying it for cohort selection, feature selection, predictive analytics, causal inference, decision support, and the development of economic models (12).

Systematic reviews of the scientific literature are essential tools for gathering and evaluating the available evidence for HTA purposes. However, the quickly growing body of original literature makes it difficult to critically evaluate, extract data and regularly update such reviews. Advances in AI systems may allow for automating a significant part of the manual work involved in evidence synthesis. This suggests that AI-based technologies have significant potential for processing both primary clinical trial data and secondary data (13) and reducing time and human burden.

Despite the immense potential of AI in health policy, there are substantial barriers that challenge HTA “doers” (e.g., HTA practitioners or researchers) to use AI to generate high-quality evidence and HTA “users” (e.g., reimbursement decision makers) to rely on AI-assisted evidence generation for decision-making (3, 14, 15). This topic is of particular importance for the Central and Eastern European (CEE) countries, as the implementation of HTA (16) and access to health databases lag behind Western European (WE) countries (17, 18); improvements in HTA are further mandated by the poorer population health in CEE than in WE countries and the corresponding need for more advanced technology across the entire healthcare sector. The digitalization of health data is also developing in CEE, where in most countries healthcare financing is less fragmented due to single payer systems. This provides the opportunity to track patient pathways, explore comparative effectiveness data and estimate resource use and costs for different diseases in a single national database (3). AI may accelerate the generation of locally digested evidence from electronic databases and reduce the gap in HTA implementation between CEE and WE countries. In addition to using databases as sources of evidence generation, AI may support the analysis of (massive) health-policy-related textual corpora. Finally, AI also has the potential to make decisions accountable, transparent, and more evidence-driven, which needs to be improved, particularly in CEE countries (16, 19).

This research was conducted as part of the HTx project. HTx is a Horizon 2020 project supported by the European Union, lasting for 5 years from January 2019.^2^ The main aim of HTx is to create a framework for the Next Generation Health Technology Assessment to support patient-centered, societally oriented, real-time decision-making on access to and reimbursement for health technologies throughout Europe (20). This initiative is consistent with and further strengthens efforts at EU level to develop a framework for joint assessments (21). This publication aims to provide recommendations to support healthcare decision-makers in more effectively integrating AI into the HTA methodologies and processes, with a particular emphasis on CEE countries. This paper does not cover AI as a tool that can aid medical diagnostics, analyse images, or predict risks since, in these applications, AI can be considered a health technology in itself. Instead, this paper explores how to better exploit the potential of AI to generate evidence for decision-making.

We have two major goals. The first is to prioritize previously identified barriers developed in the context of CEE countries (3) and the second is to propose generalizable recommendations on how to overcome the most important ones.

## 2. Methods

### 2.1. Identifying barriers

A previous work product of the HTx project, including a literature review and focus group, identified a total of 29 barriers that specifically hinder the use of AI-based evidence in HTA systems in CEE countries (3). The barriers in that study were categorized as data-related, methodological, technological, regulatory and policy-related, and human-factor-related (3). The results of the study were used as a basis to select the most important barriers for the CEE countries.

### 2.2. Selecting the most important barriers

Based on the identified barriers a survey was constructed to assess their importance for different stakeholder groups (see Supplementary file 1). Respondents were identified and contacted from the professional networks of the HTx project team members from CEE countries and other lower-income countries, where the health status of the general population is relatively poor. The survey was electronically distributed among representatives of payers, HTA organizations, academia, non-academic research institutions and consultancy companies, healthcare professionals, and health technology providers or manufacturers. Each barrier was rated using a Likert scale from 1 (very low importance) to 5 (very high importance). The results of all respondents were aggregated by calculating the average score for each barrier. Those barriers, rated to be of at least medium-high importance (with an average score 3.5 or above on the Likert scale), were considered relevant, for which recommendations should be developed.

### 2.3. Developing recommendations

#### 2.3.1. Internal brainstorming of CEE partners

After the top barriers had been selected, a series of iterative meetings were held by the CEE partners of the HTx consortium to develop recommendations to address the barriers that specifically affect CEE countries in relying on AI-based evidence during the HTA process. The Medical University of Sofia from Bulgaria and Syreon Research Institute from Hungary were the two members of the HTx consortium from CEE, and therefore they were uniquely positioned to draft problem formulation and initial recommendations.

#### 2.3.2. Workshop

The recommendations were reviewed and discussed by a wider group of experts, including HTA and reimbursement decision-makers from CEE countries and WE countries, during the workshop held on June 1, 2022 in Pula, Croatia as a pre-conference event of the annual Adriatic Pharmacoeconomic Conference. In order to explore how CEE countries could more effectively join EU-level initiatives (in the field of AI-based research and HTA cooperation), it was essential to include the perspectives of WE countries. Their role was not to assess the recommendations made by the CEE representatives, but to provide further thoughts on how best to overcome the barriers and to share their experiences with the rest of the group on some of the suggestions made. The main selection criteria of inviting workshop participants were familiarity with HTA, and health policy decisions and balancing participants based on their geographical location. Additionally, to ensure diversity of viewpoints, the experts from CEE countries had various levels of experience with HTA and the use of AI for supporting HTA.

The workshop started with a presentation (1) on the barriers to use AI in HTA by groups (data-related, technological, regulatory and policy-related and human factor related) and (2) potential recommendations how to overcome them. Participants were asked to comment on each recommendations based on their expertise, while a senior HTx researcher moderated the discussion and channeled the conclusions into recommendations. The entire discussion was audio recorded, which was summarized in a written report highlighting the key ideas arising from the workshop. After the workshop, this report was shared with participants who had the opportunity to challenge and comment on the formulated recommendations. The feedback from the co-authors and participants was then used to improve the description of the recommendations.

## 3. Results

### 3.1. Barrier ranking

A total of 77 respondents filled out the survey including 20 payer representatives, 23 HTA organization representatives, 15 academic researchers, 5 non-academic researchers or consultants, 3 healthcare professionals, and 11 experts from health technology manufacturers. Survey respondents represented 11 countries from the region, including Bulgaria, Croatia, Hungary, Kazakhstan, Poland, Republic of North Macedonia, Romania, Serbia, Slovakia, Turkey and Ukraine. The results of the ranking are summarized in Table 1.

**Table 1:** Ranking results of the survey.

Rank	Barrier	Mean Likert score	Barrier group
1	Lack of decision-makers’ expertise about the methods and use of AI driven scientific evidence	4.03	H
2	Lack of appropriate skills for applying AI methods (natural language processing, machine learning etc.) in outcomes research	3.90	H
3	Lack of adequate education to generate AI driven scientific evidence	3.90	H
4	Issues with reliability, validity and accuracy of data (e.g., due to the lack of quality assessment of data entry or self-reporting)	3.88	D
5	Lack of awareness and openness on the part of decision-makers to rely on AI-based real-world evidence	3.85	R
6	Lack of political commitment (e.g., no health digitization strategy in the country to establish relevant databases)	3.81	R
7	Multinational data collection and analysis is limited due to differences in the coding system across countries, and the lack of mapping methods to standardize the vocabulary	3.68	D
8	Lack of resources to build and maintain IT infrastructure to support AI process	3.68	T
9	Regulatory compliance issues in the process of managing a high volume of sensitive information	3.67	R
10	Analysis of multicentre data is limited due to differences in database structures across systems (e.g., electronic medical records database of different service providers)	3.63	D
11	Raw fragmented or unstructured data (e.g., electronic medical records, imaging reports), which are difficult to aggregate and analyse	3.62	D
12	High cost of improving data validity (e.g., data abstracters to evaluate unstructured data)	3.62	T
13	Systemic bias in the data (e.g., due to upcoding)	3.59	D
14	Lack of well-described patient-level health databases	3.59	D
15	Data that are relevant for research purposes (e.g., important clinical endpoints) are missing from databases or are available only on paper.	3.54	D
16	The database is incomplete to fully track patient pathways, leading to inconsistent, unreliable findings	3.49	D
17	High costs associated with securing and storing data for research purposes	3.47	T
18	Data is not transferable across countries for multinational analyses	3.46	D
19	Lack of access to patient-level databases due to data protection regulations	3.42	R
20	Lack of knowledge in data governance: data ownership and data stewardship	3.42	H
21	Lack of transparency of protocols for data collection methods	3.37	M
22	Data cleansing is not feasible	3.28	D
23	Lack of methodological transparency of deep learning models (“black box” phenomenon)	3.18	M
24	Potential bias of AI to favour some subgroups based on having more or better information	3.15	M
25	Sample size of the available databases are low (e.g., databases of health care providers are not linked)	3.13	D
26	Acceptance and consent by patients and medical professionals	3.13	R
27	Text mining and natural language processing algorithms cannot be applied due to the lack of standardized medical terms in the local language	3.12	M
28	The result of analysing complex diseases with AI is difficult to use in health economic models	3.10	M
29	Limited reproducibility due to the complexity of AI methods	3.06	M

D, data related barriers; H, human factor related barriers; M, methodological barriers; R, regulatory and policy related barriers; T, technological barriers.

Out of the 29, there were 15 barriers that respondents rated as medium to very important. These were 7 out of 11 data-related barriers, 2 out of 3 technology-related barriers, 3 out of 5 regulatory and policy-related barriers, and 3 out of 4 human factor-related barriers. None of the 6 methodological barriers were considered important enough to be included. Initial recommendations were developed for the selected barriers and presented at the workshop.

The ranking is clearly topped by “human factor related barriers,” which are the lack of expertise, skills and education (rank 1–3). Ranked 5–6 is the need for awareness and openness of decision makers to rely on real-world evidence, and the lack of their political commitment (regulatory and political barriers). In addition, data-related barriers (ranked items 4, 7, 10, 11, 13, 14, and 15) and technological barriers, including lack of resources and high costs of data validation (ranked items 8, 12) were also high priorities.

### 3.2. Workshop results

Overall, 23 experts representing 12 European countries (Bulgaria, Belgium, Hungary, Poland, Romania, Serbia, Slovakia, Slovenia, Spain, The Netherlands, UK, and Ukraine) participated in the workshop and contributed to the validation of recommendations. Participants represented different stakeholder groups at the workshop: academia (*n* = 7), HTA body (*n* = 5), patient representative (*n* = 1), payer (*n* = 4), and non-academic research institute (*n* = 6).

#### 3.2.1. Recommendations for human-factor-related barriers

Human-factor-related barriers

- Lack of decision-makers’ expertise about the methods and use of AI driven scientific evidence
- Lack of appropriate skills for applying AI methods (natural language processing, machine learning etc.) in outcomes research
- Lack of adequate education to generate AI driven scientific evidence

##### 3.2.1.1. Educating HTA “doers” and “users”

To better understand the application of AI, developing specific training materials on how to use AI for HTA to generate evidence and organizing training courses on HTA for non-AI experts and users is recommended. HTA experts are advised to rely on the high-quality training programs already available to learn and master AI methods.

##### 3.2.1.2. Establishing collaborations

If the experts involved in the HTA process lack the methodological knowledge to critically evaluate AI methods, it is advisable to cooperate with academic centers and involve experts who have the necessary depth of knowledge and can give an unbiased opinion.

Expertise can also be developed by ensuring a diverse representation of researchers from both WE and CEE countries in EU funded international collaborative projects for the development of databases and AI methods. Such joint efforts can advance the knowledge and facilitate the generalisability and transferability of the methods developed in the project.

##### 3.2.1.3. Best practice sharing

The creation or use of a virtual platform [such as the Decide Health Decision Hub (22)] for experience exchange between countries to support knowledge transfer is recommended.

As national stakeholders and decision-makers might have a limited view on what the local benefits could be, describing good practices for decision-makers about how to use AI is recommended. This can facilitate building trust towards AI-based methods.

For HTA purposes, it is advised to apply widely used AI methods in the analyses instead of custom-developed solutions, as this will ultimately improve the transferability and generalisability of the method.

#### 3.2.2. Recommendations To address regulatory and policy-related barriers

Regulatory and policy-related barriers

- Lack of awareness and openness on the part of decision-makers to rely on AI-based real-world evidence
- Lack of political commitment (e.g., no health digitization strategy in the country to establish relevant databases)
- Regulatory compliance issues in the process of managing a high volume of sensitive information

##### 3.2.2.1. Increasing awareness and political commitment

The engagement of policymakers is key to improving the regulatory environment for accessing and processing large amounts of health data. Therefore, demonstrating the advantages and usability of AI in HTA for different stakeholders is recommended by presenting use cases on efficiency improvements and international trends on the use of AI for HTA decision-making. Benchmarks can be used to compare different industrial sectors and geographical regions in terms of the contribution of AI to their economic growth and sustainability.

Health insurance data are well suited to impact major health policy decisions affecting a wider population. Therefore, advocating for greater reliance on the claims database by policymakers is recommended, which can then lead to better regulation of its use. This could also facilitate the use of the database for AI-assisted HTA. Training and exchange of experience could be organized at the policymakers’ level to better understand AI’s relevance for HTA. Attention to the applicability of AI in HTA could be further enhanced if reporting requirements for the use of AI are introduced in the HTA guidelines.

##### 3.2.2.2. Improving the management of sensitive information for AI use

As existing medical data is usually stored in silos and privacy concerns limit access to the data, AI cannot reach its full potential. This problem could be addressed by federated learning techniques that allow multiple parties to train together without the need to swap or centralize datasets, thus addressing the problems associated with sharing sensitive medical data.

Although many governments have established laws and compliance standards by which sensitive data should be used, investing in the development of anonymization and pseudonymization methods is recommended, specifically for databases that can be linked and used to generate evidence (e.g., electronic medical records, claims databases, registries). Trusted research environments, as established by NHS for England (23), can provide licensed researchers with access to essential linked, de-identified health data and thus promote the safe use of data in research. In addition, to protect patient privacy and enhance clinical research, it is advisable to facilitate the creation of synthetic medical data generated algorithmically rather than based on real events. This can increase the robustness and adaptability of AI models (24). Another approach to accessing individual patient data is to develop databases that provide individuals with free access to services on specific platforms in exchange for voluntary sharing of their health data.

Seeking feedback from regulators on the feasibility of obtaining and analysing individual patient data from health databases and assessing the data access process in a pilot study before making large-scale investments in data processing is recommended. Data management rules and standard operating procedures should be continuously improved as knowledge of AI-based analytics evolves. This can be seen as joint learning for AI users and regulators.

#### 3.2.3. Recommendations to address data-related barriers

Data-related barriers

- Issues with reliability, validity and accuracy of data (e.g., due to the lack of quality assessment of data entry or self-reporting)
- Multinational data collection and analysis is limited due to differences in the coding systems across countries, and the lack of mapping methods to standardize the vocabulary
- Analysis of multicentre data is limited due to differences in database structures across systems (e.g., electronic medical records database of different service providers)
- Raw, fragmented or unstructured data (e.g., electronic medical records, imaging reports), which are difficult to aggregate and analyse
- Systemic bias in the data (e.g., due to upcoding)
- Lack of well-described patient-level health databases
- Data that are relevant for research purposes (e.g., important clinical endpoints) are missing from databases or are available only on paper.
- It has to be emphasized that most of the recommendations below are valid for any data collection and analysis leading to evidence synthesis and are not narrowly specific for working with AI tools and methods; this can be expected as data barriers themselves are not narrowly AI-specific as well.

##### 3.2.3.1. Enhancing standardization and collaboration with data networks

To enhance multinational and multicentric analysis of data, using shared, standardized data structures and models, e.g., Observational and Medical Outcomes Partnerships Common Data Model (OMOP CDM) (25), is recommended. This requires structural mapping of health databases and terminological mapping to convert local codes (e.g., procedures) into standardized vocabularies, e.g., Systematized Nomenclature of Medicine—Clinical Terms (SNOMED CT) (26). Countries updating their local coding systems for procedures, diagnoses, laboratory results, or drugs should already consider the local introduction of internationally recognized, standardized vocabularies, for example, as proposed by the WHO (27). Such standardization also allows for federated learning, e.g., European Health Data Evidence Network (EHDEN) (28). As in most cases, it is not possible to standardize all parameters, defining the minimum common data set to be provided by the participating centers is recommended in joint studies.

##### 3.2.3.2. Managing missing and unstructured data

Checking whether the results can be used without the missing data is recommended. If, for example, results cannot be used even with imputation, it is crucial to assess whether it is worth investing resources to collect the data afterward, or how much resources and costs are needed to digitise the paper information. In case of unstructured data (e.g., text corpora), whether data on key variables are available in the text fields (e.g., as in medical reports) should be checked. If so, text mining methods (e.g., correlation, collocation, phrase frequency analysis) could be used to extract values in a structured format. Text mining methods can also be useful for efficient identification and selection of studies, extraction of data, and analysis and presentation of results from secondary sources (e.g., published literature).

##### 3.2.3.3. Using analytical and statistical approaches to address bias

In outcome research, bias in data can be mitigated by focusing on relative measures (e.g., effectiveness), using control groups, and examining whether bias may differ in the groups being compared. Tracking the patient pathway of a sample of patients prior to analysis can be useful to improve the analysis design. If the information on the hard endpoint is partially missing from the sample, the data could be censored if a sufficient number of subjects are available. If the hard endpoints are missing or unreliable, it is advisable to focus on intermediate outcomes in the analysis.

In epidemiological research, data can be cross-validated using other databases (e.g., claims data along with prescription data). It is not always necessary to stick to patient-level data, as higher-level, aggregated data can be accepted for analysis if sufficient data are available to train the algorithm and the result is meaningful for the research question (e.g., for epidemiological analysis at the district level). If AI is used to analyse secondary data from published literature, the potential publication bias (over-publication of positive study results) should be taken into account when developing algorithms for evidence synthesis (e.g., network meta-analysis).

##### 3.2.3.4. Using quality assessment tools and quality standards

Whenever possible, the following is recommended: (i) conducting data profiling to understand better the data, (ii) reviewing data entry methods, and (iii) validating data by discussing descriptive statistics with clinical experts who enter the data. The use of data quality assessment tools is recommended to measure data quality (e.g., risk of bias for secondary data) and apply quality rules. E.g., missing data rates should not exceed 20% in any field for primary data.

Further, the use of algorithms or face validity checks to help define technical definitions and rules to improve quality and usability (e.g., using a diagnosis code at least two times in a year to qualify as a chronic co-morbidity) is recommended. These could be made transparent in the description of the analysis. To facilitate data reliability, validity and accuracy, different approaches are recommended when AI is used to process primary (e.g., electronic medical records) or secondary (systematic literature reviews) data in HTA.

##### 3.2.3.5. Improving reporting

Where evidence generated with the support of AI is used for HTA, a chapter on limitations on the reliability, validity, and accuracy of the data is recommended to be a standard part of the report. The technical description of the AI-based tool used for the analysis, submitted as part of the HTA documentation, could be accompanied by an informative lay description for HTA experts. This should require no more than a competent level of knowledge of the related methods. The algorithm used by the specific AI tool needs to be transparent, refer to published sources, and in the case of using ML, the process of “training” should be reproducible.

If patient-level health databases are used for evidence collection, it is recommended that they be required to disclose scrupulously depersonalized or aggregated data in a way that supports better transferability of real-world evidence to other jurisdictions. As literature widely refers to it, evidence about what works in clinical practice should be considered as public good, especially if it was generated with use of public fund or is used for reimbursement decisions (29, 30). Standardized reporting guidelines for publication, such as GRADE (31–33) or the ISPOR and ISPE joint Task Force initiative for transparency in RWE (34) can be used to facilitate AI-assisted aggregation based on secondary data.

##### 3.2.3.6. Developing better conditions for the use of data

Improved access to data and improved conditions for data analysis can enhance the use of advanced analytical approaches to generate evidence. This could be facilitated by establishing interconnection of different databases on various levels, e.g., linking episode-based electronic medical records with longitudinal patient records and payer databases. Since claims databases consist of large, structured data sets, using these databases for HTA is encouraged, with trained staff, budgets, and technical environment to apply AI tools. To improve the structure and quality of patient-level real-world health data recorded at healthcare providers, regulatory measures could ensure standardized and validated data collection in clinical practice. Best practices in superior quality data collection could be reviewed in other countries. Publication of good local practices in international journals would help disseminate knowledge in this area.

#### 3.2.4. Recommendations to address technological barriers

Technological barriers

- Lack of resources to build and maintain IT infrastructure to support AI process
- High cost of improving data validity (e.g., data abstracters to evaluate unstructured data)

Due to the high resource requirements of using AI tools, no specific capacity should be built for a single project only. AI infrastructure and human capacities should be developed, reused and continuously upgraded in centers of excellence, taking into account economies of scale. To ensure efficient use of public resources, a sustainability plan should be submitted for all publicly funded (Horizon Europe, Innovative Health Initiative) projects related to database development or AI. International centers for generating and exchanging AI-based evidence in medicine and HTA should be established, or existing organisations should take the lead in facilitating such collaboration at the EU level.

The excessive cost of deploying and maintaining large computing capacities can be addressed by using technologies (e.g., cloud storage and server capacities) scaled to the needs of analyses performed. A higher number of projects can reduce the unit cost of storage and security (economies of scale). If improving data validity is very resource intensive, it is advisable to assess the added value of improving data validity in the first place. The expected value of perfect information (EVPI) analysis, which has already been used in HTA, can be applied to measure the expected cost of a wrong reimbursement decision due to uncertainty in data and an AI algorithm based on unreliable data. A pilot study to assess the validity of a sample of data can support the feasibility of analysis at an early stage of a project before significant resources are devoted to the costly development of learning algorithms on a large dataset.

## 4. Discussion

AI has great potential to evaluate large amounts of health data, but this has not been adequately exploited by HTA “doers” due to various issues (including data-related, methodological, technological, regulatory and policy-related, and human-factor-related barriers) (3, 35, 36). The HTA community and health policymakers could move forward by relying on new trustful methods to further improve the efficiency of reimbursement decision-making. In this regard, good practices and examples could build upon trust and reduce uncertainties for HTA bodies when considering AI among their common tools. There are many experts in statistics, informatics, and data science who are master users of AI tools, and HTA often requires processing much information to improve the evidence base and reduce reimbursement decision uncertainty. However, HTA “doers” and “users” may not be aware of how to channel this expertise and better integrate advanced AI methods into HTA processes. Our study helps to reduce this knowledge gap by providing recommendations from a diverse group of AI and HTA experts for both HTA “doers” and “users.” Within the broad field of AI, our efforts focused on using ML methods for evidence generation and synthesis in the field of HTA—as these state-of-the-art methods and tools are now increasingly available both as products and services.

The study also aimed to contribute to narrowing the gap between lower-income CEE countries and higher-income WE countries in data-driven decision-making processes, and to that end, we focused on the CEE countries to prioritize which barriers should be addressed. The recommendations do not cover all areas where improvements are needed but focus on areas where action should and can be taken soon. Although stakeholders from CEE countries ranked the barriers, the representatives of higher-income WE countries at the workshop highlighted that they also face similar challenges, so the recommendations are not only applicable in CEE countries but can be generalized to countries with more developed health systems as well.

The ranking of barriers shows that human-factor and regulatory and policy barriers were identified as the most pressing issues to overcome. But numerous data-related barriers were also identified as priorities to be addressed. The barriers categorized as ‘methodological barriers’ were not ranked highly and therefore considered only second-tier problems in implementing AI in HTA. But it might be considered that if, for instance, some of the data-related barriers are solved, methodological barriers may become more prominent because these barriers are strongly intertwined. Interestingly, some frequently cited factors such as the importance of acceptance and consent by patients and health professionals were only ranked 26 out of 29; the lack of common clinical endpoints (15), and the tracking of patient pathways (16). This result may be an indication of the balance of representation of stakeholders in the survey.

A general recommendation is to raise awareness of the benefits of AI-assisted health policymaking and encourage policymakers’ political commitment to create the regulatory and infrastructural environment and knowledge base (i.e., skilled staff) necessary to better integrate AI into HTA decision-making processes.

Several initiatives can be seen at the EU level that try to offer solutions to many data-related problems. The Joint Action Towards the European Health Data Space (TEHDAS) develops European principles for the secondary use of health data (37). DARWIN EU and EHDEN adopt common data models and establish federated data networks. The open-source OMOP CDM standardizes the structure, format, and terminology of otherwise disparate datasets, allowing the implementation of common analysis codes through a federated data network in which only codes and aggregated results are shared (38). For the coming decade, the recent publication of WHO’s new version of the International Classification of Diseases (ICD 11) and the renewed International Classification of Health Interventions (ICHI) will result in much more meaningful and detailed coding of patient data for HTA, opening up further avenues of evidence generation (39). While common data models have become more frequently used in regulatory decision-making, relatively little attention has been given to their use in (HTA) (40).

As these new initiatives can promote data-driven AI-assisted decision-making, they also provide CEE countries, if they participate, with a better position to represent their populations in pooled or federated datasets. These can potentially be used for EU-level regulatory decisions and joint HTA; therefore, it is particularly important that CEE countries join these initiatives.

Several articles have discussed the challenges with the HTA of AI-driven health interventions (2, 41, 42). They have proposed to take into account the exceptional aspects of AI-based technologies to adapt HTA frameworks to make them better suited to evaluate such technologies. However, to our knowledge, this is the first study that discusses AI in the context of generating evidence for HTA and reimbursement decisions. AI methods can generate evidence by using computer programs to discover previously unrecognizable patterns and associations in large data sets and incorporate them into predictive models. However, these results must be evaluated with the same scientific rigour that is at the heart of evidence-based medicine (EBM) and HTA. As mentioned above, to preserve the values of the scientific approach, all algorithms used by AI tools should be made transparent, refer to published sources, and, e.g., in the case of using ML, the training process should be reproducible by independent research. Traditional methods of evaluating evidence will also benefit from adding AI to better inform individualized decision-making processes (43, 44). This can pave the way towards a next generation of HTA practices but does not put into doubts the basics of HTA processes (20).

It should be noted that the use of AI, in addition to its potential, also carries risks, as AI algorithms can increase discrimination and inequity in healthcare. Underserved populations are less represented in healthcare databases, and therefore AI algorithms based on such data may reinforce these patterns due to their learning methods (12). Therefore, decision-makers must be aware of the potential for bias and proactively seek to overcome it.

The study has some limitations. Although the total number of respondents (*n* = 77) was satisfactory, the representation of patients and health professionals was low in the survey. This may have resulted in some potentially important factors for these groups being ranked lower. In addition, the threshold for taking barriers into account (3.5) was based on the average score of all respondents, which may have excluded items that were only very important for certain groups. Our recommendations are primarily based on the opinions of a relatively small group of experts, which is the main limitation. However, the fact that the experts who participated in the deliberation process represented countries with a range of economic backgrounds and health systems strengthens our findings’ generalizability to CEE countries.

Overall, our recommendations should only be seen as a first step in a multi-stakeholder dialog on how to better integrate AI methods into HTA practices and how to translate proposals to address existing barriers into action. As the use of AI technology spreads and awareness of its use and potential pitfalls becomes more widespread and deeper, there may be a need to reassess the barriers to its use in HTA conducting and to look more broadly at the barriers that have not been addressed in this study.

## 5. Conclusion

In the field of HTA, the great potential of AI to support evidence generation and evaluation has not yet been sufficiently realized. Raising awareness of the intended and unintended consequences of AI-based methods and encouraging political commitment from policymakers is necessary to upgrade the regulatory and infrastructural environment and knowledge base necessary to better integrate AI into HTA-based decision-making processes.

## Data availability statement

The original contributions presented in the study are included in the article/Supplementary material, further inquiries can be directed to the corresponding author.

## Ethics statement

Ethical review and approval was not required for the study on human participants in accordance with the local legislation and institutional requirements. Written informed consent from the participants was not required to participate in this study in accordance with the national legislation and the institutional requirements.

## Author contributions

AZ, KT, LB, BN, ZP and ZK contributed to the initial design and concept of this research. AZ wrote the initial draft of the paper. KT, GP, DD, WG IG, RH, SK OP, and ZK provided significant editing. GP, MC, DD, WG, IG, RH, SK, LL, ZM, OP, AS, MM, TT, and SZ actively took part in the discussions on finalizing the recommendations and critically reviewed the manuscript prior to submission. All authors contributed to the article and approved the submitted version.

## Funding

This project has received funding from the European Union’s Horizon 2020 research and innovation programme under Grant Agreement No. 82516.

## Conflict of interest

The authors declare that the research was conducted in the absence of any commercial or financial relationships that could be construed as a potential conflict of interest.

## Publisher’s note

All claims expressed in this article are solely those of the authors and do not necessarily represent those of their affiliated organizations, or those of the publisher, the editors and the reviewers. Any product that may be evaluated in this article, or claim that may be made by its manufacturer, is not guaranteed or endorsed by the publisher.

## Footnotes

^1^ https://www.coe.int/en/web/artificial-intelligence/ai-covid19

^2^ www.htx-h2020.eu
