# Supplementary material for: Recommendations to overcome barriers to the use of artificial intelligence-driven evidence in health technology assessment
Source: Front Public Health. 2023 Apr 26;11:1088121. doi: 10.3389/fpubh.2023.1088121 (PMC10171457; doi:10.3389/fpubh.2023.1088121)
Supplement: Supplementary file 1 [file Table_1.DOCX]

**Supplementary material 1 - HTx AI Implementation Barriers Survey**

Artificial intelligence (AI) is used to mimic human cognition in analyzing, presenting and understanding complex data. In health care, with the increasing use of information systems and access to large amounts of data, the application of AI tools (e.g. machine learning, deep learning, text mining or natural language processing) might facilitate evidence-based decision-making.

Specifically, in the field of HTA, researchers can rely on health systems data such as administrative claims or electronic health records to generate evidence on health outcomes to support the decisions of policy makers and inform patients about the utilization practice, effectiveness or costs of technologies. However, the opportunity of considering AI-driven evidence in HTA are not without challenges.

**Please rank the barriers to use and rely on artificial intelligence (AI) driven scientific evidence in the HTA process on the scale from 1 to 5: choose 1 to indicate the barrier with the lowest importance and 5 for those with the highest importance**

| **Barriers** | | **Very low importance (1)** | **Low importance (2)** | **Medium importance (3)** | **High importance (4)** | **Very high importance (5)** |
| --- | --- | --- | --- | --- | --- | --- |
| Data related barriers | Issues with reliability, validity and accuracy of data (e.g. due to the lack of quality assessment of data entry or self-reporting) |  |  |  |  |  |
|  | Systemic bias in the data (e.g. due to upcoding) |  |  |  |  |  |
|  | Raw fragmented or unstructured data (e.g. electronic medical records, imaging reports), which are difficult to aggregate and analyze |  |  |  |  |  |
|  | Data cleansing is not feasible |  |  |  |  |  |
|  | Analysis of multicenter data is limited due differences in database structures across systems (e.g. electronic medical records database of different service providers) |  |  |  |  |  |
|  | Lack of well-described patient level health databases |  |  |  |  |  |
|  | Multinational data collection and analysis is limited due to differences in coding system across countries, and the lack of mapping methods to standardize the vocabulary |  |  |  |  |  |
|  | Data that are relevant for research purposes (e.g. important clinical endpoints) are missing from databases or are available only on paper. |  |  |  |  |  |
|  | Data is not transferable across countries for multinational analyses |  |  |  |  |  |
|  | The database is incomplete to fully track patient pathways, leading to inconsistent, unreliable findings |  |  |  |  |  |
|  | Sample size of the available databases are low (e.g. databases of health care providers are not linked) |  |  |  |  |  |
| Methodological barriers | Potential bias of AI to favor some subgroups based on having more or better information |  |  |  |  |  |
|  | Lack of transparency of protocols for data collection methods |  |  |  |  |  |
|  | Text mining and natural language processing algorithms cannot be applied due to the lack of standardized medical terms in the local language |  |  |  |  |  |
|  | Limited reproducibility due to the complexity of the methods |  |  |  |  |  |
|  | Lack of methodological transparency of deep learning models (“black box” phenomenon) |  |  |  |  |  |
|  | The result of analyzing complex diseases with AI is difficult to use in health economic models |  |  |  |  |  |
| Technological barriers | Lack of capacity to build and maintain IT infrastructure to support AI process |  |  |  |  |  |
|  | High costs associated with securing and storing data for research purposes |  |  |  |  |  |
|  | High cost of improving data validity (e.g. data abstracters to evaluate unstructured data) |  |  |  |  |  |
| Regulatory and policy related barriers | Regulatory compliance issues in the process of managing high volume of sensitive information |  |  |  |  |  |
|  | Lack of awareness and openness on the part of decision-makers to rely on AI based real-world evidence |  |  |  |  |  |
|  | Lack of political commitment (e.g. no health digitization strategy in the country to establish relevant databases) |  |  |  |  |  |
|  | Lack of acceptance and consent by patients and medical professionals |  |  |  |  |  |
|  | Lack of access to patient-level databases due to data protection regulations |  |  |  |  |  |
| Human factor related barriers | Lack of knowledge in data governance: data ownership and data stewardship |  |  |  |  |  |
|  | Lack of appropriate skills for applying AI methods (natural language processing, machine learning etc.) in outcomes research |  |  |  |  |  |
|  | Lack of adequate education to generate AI driven scientific evidence |  |  |  |  |  |
|  | Lack of decision-makers’ expertise about the methods and use of AI driven scientific evidence |  |  |  |  |  |
